# A ten-year review of ESBL and non-ESBL *Escherichia coli* bloodstream infections among children at a tertiary referral hospital in South Africa

**DOI:** 10.1371/journal.pone.0222675

**Published:** 2019-09-24

**Authors:** Oliver Ombeva Malande, James Nuttall, Vashini Pillay, Colleen Bamford, Brian Eley

**Affiliations:** 1 Paediatric Infectious Diseases Unit, Red Cross War Memorial Children’s Hospital, Cape Town, South Africa; 2 Department of Paediatrics and Child Health, University of Cape Town, Cape Town, South Africa; 3 National Health Laboratory Service, Groote Schuur Hospital, Cape Town, South Africa; 4 Division of Microbiology, University of Cape Town, Cape Town, South Africa; University of Maryland School of Medicine, UNITED STATES

## Abstract

**Introduction:**

There are few studies describing *Escherichia coli* (*E*. *coli*) bloodstream infection (BSI) among children in Africa, yet *E*.*coli* is increasing in importance as a cause of antibiotic resistant infection in paediatric settings.

**Methods:**

In this retrospective, descriptive study aspects of *E*. *coli* BSI epidemiology are described over a 10-year period including incidence risk, risk factors for extended-spectrum β-lactamase (ESBL)-producing *E*. *coli* BSI, antibiotic susceptibility of the bacterial isolates and outcome including risk factors for severe disease.

**Results:**

There were 583 new *E*. *coli* BSI episodes among 217,483 admissions, an overall incidence risk of 2.7 events/1,000 hospital admissions. Of 455 of these *E*. *coli* BSI episodes that were analysed, 136 (29.9%) were caused by ESBL-producing isolates. Risk factors for ESBL-producing *E*. *coli* BSI included hospitalization in the 28-day period preceding *E*. *coli* BSI episodes, having an underlying chronic illness other than HIV infection at the time of the *E*. *coli* BSI and having a temperature of 38° Celsius or higher at the time of the *E*. *coli* BSI. None of the *E*. *coli* isolates were resistant to carbapenems or colistin. The mortality rate was 5.9% and admission to the intensive care unit was required in 12.3% of BSI episodes. Predictors of severe disease included age less than 1 month, hospitalization in the 28-day period preceding *E*. *coli* BSI and BSI without a definable focus.

**Conclusions:**

These findings extend our understanding of *E*. *coli* BSI in a sub-Saharan African setting, provide useful information that can guide empiric treatment choices for community- and hospital-acquired BSI and help inform prevention strategies.

## Introduction

Although *Escherichia coli* (*E*. *coli*) is an important cause of bloodstream infection (BSI), there are few studies that have described the epidemiology of this infection in children who live in low- and middle-income countries. [1] Recent research has shown that *Klebsiella pneumoniae* and *E*. *coli*, both species of the family Enterobacteriaceae, are the most prevalent gram-negative pathogens isolated from children with BSI in Cape Town. [2, 3, 4] Furthermore, *E*. *coli* can cause community-acquired or hospital-acquired BSI in children and adults. [5, 6]

Multi-drug resistance caused by extended-spectrum β-lactamase (ESBL) production among Enterobacteriaceae is a global concern in paediatric and adult healthcare settings. Extended-spectrum β-lactamases are enzymes that (1) hydrolyse oxyiminocephalosporins (e.g. ceftriaxone and ceftazidime) and monobactams, (2) do not inactivate the cephamycins (e.g. cefoxitin) and carbapenems, and (3) are inhibited by β-lactamase inhibitors such as clavulanic acid and tazobactam. *E*. *coli* and *K*. *pneumoniae* are two of the main ESBL-producing microorganisms in clinical practice. [7, 8] The most frequently encountered ESBLs in clinical isolates are members of the SHV, TEM and CTX-M families. Extended-spectrum β-lactamases from all three families have been identified in Enterobacteriaceae species cultured from clinical sites in South African patients. [9, 10] Patients with infections caused by ESBL-producing Enterobacteriaceae may experience poor outcome because of delays in initiating effective antimicrobial therapy and/or lack of availability of limited antibiotic options, resulting in high mortality.[11] The World Health Organization (WHO) has included cephalosporin-resistant Enterobacteriaceae in its group of priority 1 pathogens, for which research and development of new antibiotic options are urgently required. [12]

A recent study described the epidemiology of *K*. *pneumoniae* BSI in children treated at Red Cross War Memorial Children’s Hospital (RCWMCH) in Cape Town over a six-year period [11]. In the present complementary study we describe the clinical presentation, treatment and outcome of *E*. *coli* BSI, risk factors for ESBL-producing *E*. *coli* BSI (ESBL-ECBSI), antibiotic susceptibility of *E*. *coli* isolates, and predictors of severe disease in children at RCWMCH over a ten-year period.

## Methods

### Study design and setting

This retrospective, descriptive study was conducted at RCWMCH in children hospitalised with *E*. *coli* BSI between 1 January 2005 and 31 December 2014. Red Cross War Memorial Children’s Hospital in Cape Town, South Africa, serves as a major tertiary-level referral centre for sick children aged 0 to 13 years.

### Data collection

Relevant demographic and clinical data were extracted from the medical records. HIV test results and antibiotic susceptibility results were obtained from the NHLS laboratory database and also entered on the data collection forms.

### Microbiological procedures

Microbiological testing of blood culture specimens was performed at the Medical Microbiology Laboratory of the NHLS, based at Groote Schuur Hospital, using the BACTEC 9240 automated blood culture system (Becton Dickinson, Maryland, USA) between 2005 and 2012, and the BacT/ALERT automated blood culture system ((bioMererieux Inc., Durham, NC, USA) from 2013–2014. Identification of *E*. *coli* and antibiotic susceptibility testing was carried out on the automated Vitek^®^2 system (bioMérieux, Inc., France) using Vitek^®^2 ID-GNB and AST-N255 cards. Susceptibility results were interpreted according to the Clinical Laboratory Standards Institute (CLSI) criteria for the relevant years from 2005–2014. [13–22]

The presence of ESBLs was determined by the Vitek® 2 Advanced Expert system, but due to limitations of the laboratory information system, the presence of an ESBL was not routinely reported in a standardized manner on the laboratory report. The laboratory, in line with the contemporary national practice, continued to report all ESBL- producing *Enterobacteriaceae* as resistant to all cephalosporins, apart from cefoxitin, even after the changes to reporting of cephalosporin susceptibility in ESBL-producing organisms introduced by CLSI in 2010.

#### Definitions

*Hospital-acquired Escherichia coli bloodstream infection* (HA-ECBSI): *Escherichia coli* bloodstream infection detected 48 hours or more after hospital admission and not incubating at the time of hospitalization, adapted from reference [23].

*Healthcare-associated Escherichia coli bloodstream infection* (HCA-ECBSI): *Escherichia coli* bloodstream infection detected within 48 hours of hospital admission in children who have had contact with the healthcare service including admission to an intermediate care facility during the one year period preceding hospitalization. An intermediate facility provides step-down care for children recovering from acute illness, usually for an average duration of 6 weeks, adapted from reference [23].

*Community-acquired Escherichia coli bloodstream infection* (CA-ECBSI): *Escherichia coli* bloodstream infection detected within 48 hours of hospital admission without previous contact with the healthcare service, adapted from reference [23].

*ESBL-producing Escherichia coli* (ESBL-EC): Based on local laboratory reporting practices as described above, ESBL-production was assumed if *Escherichia coli* isolates were reported as resistant to all cephalosporins, apart from cefoxitin.

*HIV infection*: A positive HIV DNA PCR result confirmed by either a HIV RNA PCR or repeat HIV DNA PCR test in a child <18 months old, or 2 positive serological test results (HIV Rapid test or HIV ELISA) or a positive HIV DNA PCR result confirmed by either a HIV RNA PCR or repeat HIV DNA PCR test in a child >18 months old was regarded as HIV-infected.[24]

*Nutritional status*: moderate and severe underweight were defined as weight-for-age z score (WAZ) between -2 and -3 standard deviations (SD) below the median WHO growth reference standards, and a WAZ < -3 SD respectively. Weight-for-age z score was derived from bodyweight at the time of the BSI episode using the WHO anthropometric calculator.[25]

*Antibiotic exposure in the preceding 12 months*: Exposure to an antibiotic for 48 hours or more during the 12-month period preceding the development of *E*. *coli* BSI *Exposure to 2 or more selective antibiotics/antibiotic classes in the preceding 12 months*: Exposure to 2 or more selective antibiotics of the 3^rd^ generation cephalosporins, fluroquinolones, carbapenems, aminoglycosides and piperacillin-tazobactam, each for a period of 48 hours or more during the 12-month period preceding the development of *E*. *coli* BSI *Successful treatment*: Recovery after treatment with antibiotics to which the isolate was susceptible.

*Coagulopathy*: An elevated out of age range prothrombin time of ≥2 seconds or an activated partial thromboplastin time of ≥60 seconds or a depressed fibrinogen level of <2 μmol/L[26,27]

*Severe disease*: Because of the low mortality recorded in this study, we were unable to evaluate predictors of death. Instead we adopted the approach of a previous study that explored predictors of severe disease. In this study we used the same definition of severe disease, namely, *E*. *coli* BSI requiring treatment in the intensive care unit (ICU) and / or death during an *E*. *coli* BSI episode. [1]

### Statistical analysis

The data was entered into Epidata version 3.1, analysed in Stata version 12.0 (StataCorp, College Station, Texas). Continuous variables were compared using the Wilcoxon rank-sum test for independent samples, and categorical variables were evaluated using the chi-square test or unadjusted Odds ratio and 95% confidence intervals (CI). For the Wilcoxon rank-sum and chi-square tests a p value of <0.05 was considered statistically significant.

Univariable analyses and multivariable logistic regression were used to identify risk factors associated with ESBL-ECBSI and severe disease. All multivariable logistic regression models were build by stepwise backward selection, incorporating variables which on univariable analyses had a p value <0.10. The results of the multivariable logistic regression models were expressed as adjusted Odds ratio (aOR) and 95% CI. Two different sets of analyses were performed. Firstly, in exploring factors associated with ESBL-ECBSI, ESBL-ECBSI was treated as the outcome or dependant variable. Potential risk factors evaluated in this analysis included gender, age category, HIV status, preceding hospitalization, underweight, temperature ≥38° Celsius, selective or multiple antibiotic exposure in the preceding 12 months, residency in an intermediate level health care facility, prolonged (>30 days) immunosuppressive therapy and underlying chronic illness other than HIV infection. The second set of analyses explored predictors of severe disease. In these analyses ESBL-ECBSI was treated as an exposure or independent variable along with other variables including gender, age category, hospitalization in the preceding 28-day period, underweight, HIV status, prolonged exposure to immunosuppressive therapy, underlying chronic disease other than HIV infection, hospital-acquired *E*. *coli* BSI and BSI without a definable focus. Both sets of analyses were repeated with the recurrent *E*. *coli* BSI episodes excluded from the dataset, to determine whether these recurrent episodes skewed the results of the multivariable logistic regression models.

### Ethical considerations

The study was conducted in accordance with the Declaration of Helsinki. It was approved by the Human Research Ethics Committee, Faculty of Health Sciences, University of Cape Town, reference number: HREC REF 699/2014, and the Research Committee of RCWMCH. Patient consent was not obtained because the data was collected and analysed retrospectively.

## Results

### Study participants

The medical microbiology laboratory database of the National Health Laboratory Service (NHLS) was searched and 647 positive *E*. *coli* blood culture results from children treated at RCWMCH were identified during the ten-year study period. Among these blood culture results, 64 repeat blood cultures performed within 30 days of diagnosing *E*. *coli* BSI on the initial blood culture, and yielding an *E*. *coli* isolate with a similar antibiogram to the initial isolate, were regarded as part of the same BSI episode and therefore excluded. The remaining 583 *E*. *coli* blood culture results represented 583 unique *E*. *coli* BSI episodes and were thus used to calculate incidence risk.

**Fig 1:**
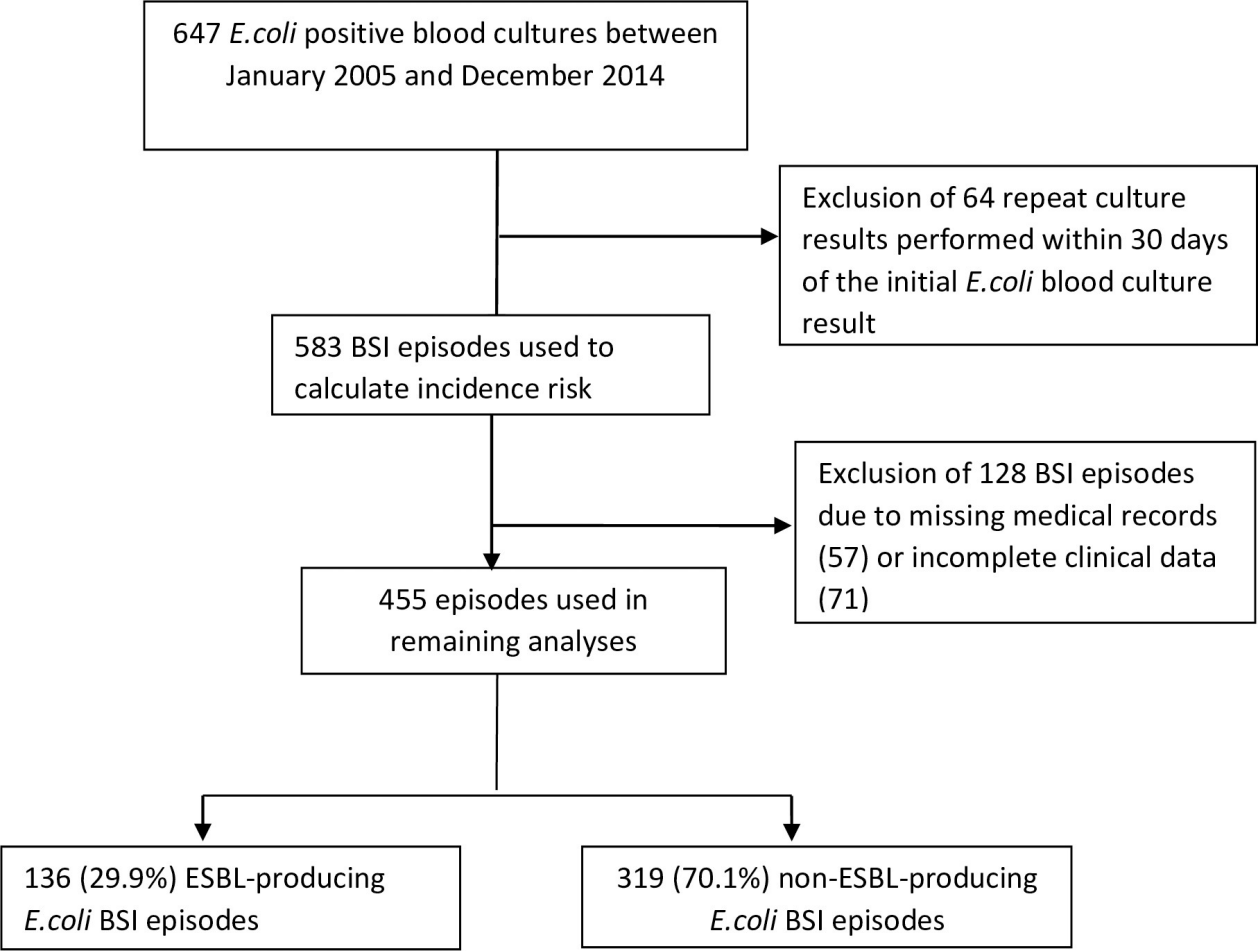
Selection of BSI episodes for data analysis.

The medical records of the children were requested from the records department at RCWMCH to complete the clinical data extraction. Unfortunately, 57 of these records could not be found and a further 71 had incomplete data relating to the *E*. *coli* BSI episodes. These *E*. *coli* BSI episodes were excluded from further analysis. Thus clinical data of the remaining 455 *E*. *coli* episodes from 443 children, and microbiology data of 455 initial *E*. *coli* isolates relating to the 455 *E*. *coli* BSI episodes (one isolate per *E*. *coli* BSI episode) were used in the remaining analyses. Twelve of the patients each experienced 2 separate *E*.*coli* BSI episodes (recurrent *E*. *coli* BSI) during the study period. One hundred and thirty six of the 455 BSI episodes (29.9%) were caused by cephalosporin-resistant *E*. *coli* isolates and assumed to be ESBL-producing isolates (Fig 1).

### Risk of *E*.*coli* BSI

**Fig 2:**
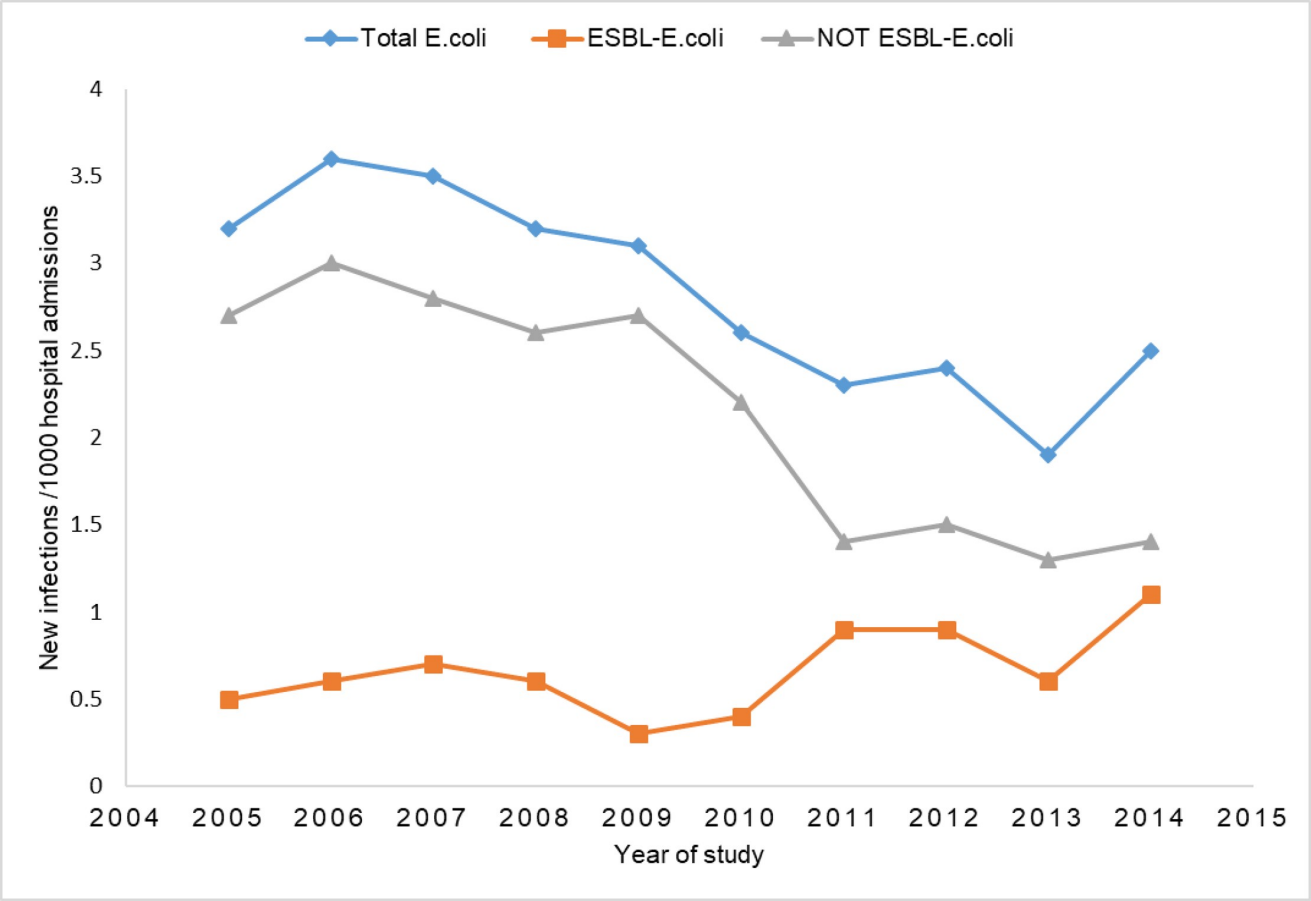
Annual incidence risk of *E*. *coli* bloodstream infection per 1000 hospital admissions at RCWMCH, January 2005 –December 2014.

During the study period 583 new *E*.*coli* BSI episodes were identified; 138/583 (23.7%) were caused by ESBL-producing isolates and 445/583 (76.3%) by non-ESBL-producing isolates. During this period there were 217,483 admissions to RCWMCH. The incidence risks over the entire study period were 2.7 *E*. *coli* BSI episodes per 1,000 hospital admissions, 0.6 ESBL-ECBSI episodes / 1,000 hospital admissions and 2.1 non-ESBL-producing *E*. *coli* BSI episodes / 1,000 hospital admissions. The annual risk of *E*. *coli* BSI declined from 3.2 episodes / 1,000 hospital admissions in 2005 to 2.5 episodes / 1,000 hospital admissions in 2014. A similar trend was observed in non-ESBL-producing *E*. *coli* annual BSI risk, decreasing from 2.7 episodes / 1,000 hospital admissions in 2005 to 1.4 episodes / 1,000 hospital admissions in 2014. By contrast, the annual risk of ESBL-ECBSI episodes increased over the study period from 0.5 episodes / 1,000 hospital admissions in 2005 to 1.1 episodes / 1,000 hospital admissions in 2014, with most of this increase occurring from 2010 onwards (Fig 2)

### Patient characteristics at the time of *E*. *coli* BSI

The median age (interquartile range) did not differ significantly in children with ESBL-ECBSI episodes compared to those with non-ESBL-producing *E*. *coli* BSI episodes, 7 (2, 16) months versus 8 (2, 18) months, p = 0.68. Table 1 summarizes baseline characteristics of the 455 participants at the time of each unique BSI episode and disaggregates the information according to the phenotype of *E*.*coli* causing the BSI.

**Table 1:** Patient characteristics at the time of *E*. *coli* bloodstream infection episodes.

DESCRIPTION	ESBL-producing *E*. *coli* episodes N = 136 n (%)	Non-ESBL-producing *E*. *coli* episodes N = 319 n (%)	Univariate OR (95% CI)
**Male gender**	73 (53.7)	196 (61.4)	0.7 (0.5, 1.1)
**Male: female ratio**	1.2:1	1.6:1	
**Age category**			
≤12 months	92 (67.7)	212 (66.5)	1.1 (0.7, 1.6)
13–60 months	30 (22.1)	79 (24.8)	
>60 months	14 (10.3)	28 (8.8)	
**HIV status**			
Infected	23 (16.9)	51 (16.0)	1.1 (0.6, 1.8)
Not infected	96 (70.6)	233 (73.0)	0.9 (0.6, 1.4)
Status unknown	17 (12.5)	35 (11.0)	1.2 (0.6, 2.2)
**Hospitalization in the preceding 12 months**	98 (72.1)	102 (32.0)	5.5 (3.5, 8.5)
**Hospitalization in preceding 28 days**	97 (71.3)	90 (28.2)	6.3 (4.1, 9.9)
**Underweight for age**			
Moderate (WAZ <-2 to >- 3 SD)	24 (17.6)	52 (16.3)	1.1 (0.6, 1.9)
Severe (WAZ <-3 SD)	51 (37.5)	90 (28.2)	1.5 (0.9, 2.3)
**Temperature ≥38** ^**o**^ **C**	119 (87.5)	246 (77.1)	2.1 (1.2, 3.7)
**Selective antibiotic exposure in the preceding 12 months**			
3^rd^ generation cephalosporin (IV)	54 (39.7)	59 (18.5)	2.9 (1.9, 4.5)
Fluoroquinolones (IV or oral)	45 (33.1)	41 (12.9)	3.4 (2.1, 5.4)
Carbapenems (IV)	58 (42.7)	52 (15.5)	3.8 (2.4, 6.0)
Aminoglycosides (IV)	96 (70.6)	98 (30.7)	5.4 (3.5, 8.4)
Piperacillin-tazobactam (IV)	71 (52.2)	58 (18.2)	4.9 (3.2, 7.6)
**Exposure to 2 or more selective antibiotics/antibiotic classes in the preceding 12 months**^**•**^	90 (28)	90 (66)	5.0 (3.2, 7.7)
**Currently resident in an intermediate level health care facility**	8 (5.9)	3 (0.9)	6.6 (1.7, 25.2)
Immunosuppressive therapy for >30 days*	26 (19.1)	23 (7.2)	3.0 (1.7, 5.6)
**Underlying chronic illness other than HIV infection**	32 (23.5)	22 (6.9)	4.2 (2.3, 7.5)

ESBL = extended-spectrum beta lactamase-producing; IQR = interquartile range; BSI = bloodstream infection; WAZ = weight-for-age Z score; SD = standard deviation, IV = intravenous

• Selective antibiotics/antibiotic classes restricted to 3^rd^ cephalosporins, fluroquinolones, carbapenems, aminoglycosides and piperacillin-tazobactam

* includes all forms of immunosuppressive therapy, and glucocorticosteroids use for the preceding 3 months.

### Risk factors for ESBL-producing *E*. *coli* BSI

On multivariable logistic regression analysis, hospitalization in the 28-day period preceding the *E*. *coli* BSI episode, exposure to intravenous piperacillin-tazobactam during the preceding 12 months, residency in an intermediate health care facility, having an underlying chronic illness other than HIV infection at the time of the *E*. *coli* BSI, and having a temperature of 38° Celsius or higher at the time of the *E*. *coli* BSI were significant risk factors for the acquisition of ESBL-ECBSI (Table 2, model 1). When the 12 recurrent *E*. *coli* BSI episodes were removed from the multivariable logistic regression model, these risk factors remained statistically significant with the exception of residency in an intermediate health care facility (Table 2, model 2). In a separate multivariable logistic regression model, exposure to 2 or more selective antibiotics / antibiotic classes in the12-month period preceding *E*. *coli* BSI was not shown to be a risk factor for ESBL-ECBSI.

**Table 2:** Risk factors of ESBL *E*. *coli* bloodstream infection determined by multivariable logistic regression.

Variable	^1^Model 1	^2^Model 2
	Adjusted Odds ratio (95% CI)	Adjusted Odds ratio (95% CI)
Hospitalization in the preceding 28-day period	3.8 (2.2–6.7)	3.6 (2.1–6.4)
Exposure to 3^rd^ generation cephalosporins in the preceding 12 months	1	1
Exposure to IV or oral fluoroquinolones in the preceding 12 months	1	1
Exposure to IV carbapenems in the preceding 12 months	1	1
Exposure to IV aminoglycosides in the preceding 12 months	1	1
Exposure to IV piperacillin-tazobactam in the preceding 12 months	2.0 (1.1–3.5)	1.8 (1.0–3.2)
Currently resident in an intermediate health care facility	4.5 (1.02–20.0)	4.0 (0.9–17.4)
Immunosupressive therapy for >30 days*	1	1
Underlying chronic illness other than HIV infection	2.2 (1.1–4.2)	2.1 (1.1–4.0)
Temperature ≥38^o^ Celsius	1.9 (1.0–3.6)	1.9 (1.0–3.6)

^1^Model 1: evaluated risk risk factors for ESBL-producing *E*. *coli* BSI using all 455 episodes i.e. 136 ESBL-producing *E*. *coli* BSI episodes and 319 non-ESBL-producing *E*. *coli* BSI episodes

^2^Model 2: evaluated risk risk factors for ESBL-producing *E*. *coli* BSI using 443 episodes i.e. 136 ESBL-producing *E*. *coli* BSI episodes and 307 non-ESBL-producing *E*. *coli BSI* episodes. In this model the 12 recurrent *E*. *coli* BSI episodes were removed from the dataset.

* includes all forms of immunosuppressive drugs and glucocorticosteroids use for more than 3 months; ESBL = extended-spectrum beta lactamase-producing; 95% CI = 95% confidence interval; IV = intravenous.

### Clinical manifestations

The majority of ESBL-producing *E*. *coli* isolates were hospital-acquired or healthcare-associated. By contrast many of the non-ESBL-producing isolates were community-acquired. Bloodstream infection without a definable site of infection was the major infection type. Of all the *E*. *coli* BSI episodes with definable infection sites, urinary tract infection and gastroenteritis were the common infections (Table 3).

**Table 3:** Clinical spectrum, treatment and outcome of *E*. *coli* BSI.

Description	ESBL-producing *E*. *coli* episodes N = 136 n (%)	Non-ESBL-producing *E*. *coli* episodes N = 319 n (%)
**Infection type**		
Hospital-acquired	71(52.2)	58 (18.2)
Community-acquired	24 (17.7)	181 (56.7)
Healthcare-associated	41(30.1)	80 (25.1)
**Infection site**		
BSI with no definable focus	103 (75.7)	185 (58.0)
UTI/Pyelonephritis	27 (19.9)	120 (37.6)
Meningitis	1 (0.7)	1 (0.3)
Septic arthritis/osteomyelitis	2 (1.5)	0
Gastroenteritis	3 (2.2)	13 (4.1)
**Empiric antibiotic treatment**		
Ampicillin & gentamicin	15 (11.1)	51 (16.0)
Amoxicillin-clavulanic acid	2 (1.5)	26 (8.2)
Ceftriaxone or cefotaxime	48 (35.3)	180 (56.4)
Piperacillin-tazobactam & amikacin	49 (36.0)	44 (13.8)
Ciprofloxacin	22 (16.2)	18 (5.6)
**Definitive antibiotic treatment**		
Amoxicillin-clavulanic acid	0	11 (3.4)
Ceftriaxone or cefotaxime	0	182 (57.1)
Gentamicin	0	30 (9.4)
Piperacillin-tazobactam & amikacin	30 (22.1)	49 (15.4)
Ciprofloxacin	0	18 (5.6)
Imipenem	1 (0.7)	1 (0.3)
Ertapenem	29 (21.3)	14 (4.4)
Meropenem	76 (55.8)	14 (4.4)
**Outcome**		
Coagulopathy	22 (16.2)	24 (7.5)
Required ICU admission	26 (19.1)	30 (9.4)
Successfully treated	121 (89.0)	289 (90.6)
Death	11 (8.1)	16 (5.0)
Transferred out*	4 (2.9)	14 (4.4)

* *E*. *coli* episodes were initially managed at the short stay ward of Red Cross War Memorial Children’s Hospital but the corresponding patients were transferred to a primary or secondary hospital to complete their antibiotic therapy; ESBL = extended-spectrum beta lactamase-producing; ICU = intensive care unit; BSI = bloodstream infection; UTI = urinary tract infection

### Antibiotic susceptibility

The median (IQR) time from admission to a positive blood culture was 1(1–3) days, range: 1–80 days. The antibiotic susceptibilities of ESBL-producing and non-ESBL-producing *E*. *coli* isolates are summarized in Table 4. Whereas 45% (58/129) of HA-ECBSI, 83.4% (171/205) of CA-ECBSI and 67.8% (82/121) of HCA-ECBSI isolates were susceptible to the combination of ampicillin plus gentamicin, 81% (85/105) of HA-ECBSI, 94.4% (168/178) of CA-ECBSI and 87.3%(89/102) of HCA-ECBSI isolates were susceptible to the combination of piperacillin-tazobactam plus amikacin.

**Table 4:** Antibiotic susceptibility of extended-spectrum beta lactamase-producing and non- extended-spectrum beta lactamase -producing *E*. *coli* isolates.

Antibiotic	Number (%) susceptible isolates	
	ESBL-producing *E*. *coli* episodes	Non-ESBL-producing *E*. *coli* episodes
Cotrimoxazole	13/123 (9.6)	50/319 (15.7)
Ampicillin or amoxicillin	0/136(0)	54/319(16.9)
Ampicillin & gentamicin	28*/136 (20.6)	283*/319 (88.7)
Amoxicillin-clavulanic acid	16/132 (12.1)	228/318 (71.7)
Ciprofloxacin	97/136 (71.3)	311/319 (97.5)
Cefuroxime	0/136 (0)	319 (100.0)
Ceftriaxone/cefotaxime	0/136 (0)	319 (100.0)
Ceftazidime	0/136 (0)	319 (100.0)
Cefepime	0/136 (0)	319 (100.0)
Gentamicin	28/136 (20.6)	283/319 (88.7)
Amikacin	108/136 (79.4)	307/317 (96.8)
Piperacillin-tazobactam	79/102 (77.5)	278/284 (97.9)
Piperacillin-tazobactam & amikacin	72^ǂ^/102 (70.6)	270^ǂ^/283 (95.4)
Imipenem	136/136 (100)	319/319 (100.0)
Meropenem	136/136 (100)	319/319 (100.0)
Ertapenem	122/122 (100)	226/226 (100.0)
Colistin	136/136 (100)	319/319 (100.0)
Tobramycin	13/109 (11.9)	274/286 (95.8)

*These isolates were susceptible to ampicillin or gentamicin, or ampicillin and gentamicin.

^ǂ^ These isolates were susceptible to piperacillin-tazobactam or amikacin, or piperacillin-tazobactam and amikacin

Some of the antibiotic susceptibility results were not reported by the laboratory, hence the variation in the denominators; ESBL = extended-spectrum beta lactamase-producing

### Antibiotic therapy

The median (IQR) duration of antibiotic therapy was 9 (5–14) days. The median (IQR) duration of antibiotic therapy was significantly longer for ESBL-ECBSI than non- ESBL *E*. *coli* BSI episodes, 10 (7–14) days vs 7 (5–14) days, p<0.001. Empiric and definitive antibiotic treatment is summarized in Table 3. During most of the 10 year study period, piperacillin-tazobactum and amikacin combination was the empiric choice for suspected hospital acquired infection. Third generation cephalosporin therapy was the predominant choice for both empiric and definitive treatment, while a carbapenem was the definitive antibiotic option in 77% of all ESBL-ECBSI episodes.

### Outcome of *E*.*coli* BSI

The median (IQR) total duration of hospitalization was 10 (5–20) days, and significantly longer for ESBL-ECBSI than non- ESBL *E*. *coli* BSI episodes, 15 (10–25) days vs 8 (5–15) days, p<0.01. While 90% of the *E*. *coli* BSI episodes were successfully treated, the outcome of 18 episodes could not be determined because the patients associated with these episodes were transferred to level 1 or 2 hospitals to complete antibiotic therapy (Table 3). During the study period, 27 children died within 30 days of the onset of the *E*. *coli* BSI. Although mortality rate was higher in ESBL-ECBSI episodes this was not statistically significant, 8.1% vs 5.0%, p = 0.29. Of the 106 ESBL E. coli BSI episodes treated with a carbapenem, 11 (10,4%) resulted in death, four of these 11 episodes had initially been treated with piperacillin-tazobactam plus amikacin before changing to definitive carbapenem therapy, while 8 of the 30 (26.7%) ESBL E. coli BSI episodes treated with piperacillin-tazobactam plus amikacin died, p = 0.03.The clinical course of 10% of E coli BSI episodes was complicated by coagulopathy and 12% required admission to the ICU for advanced care and/or monitoring. A significantly higher proportion of ESBL-ECBSI than non- ESBL *E*. *coli* BSI episodes required ICU admission, 19.1% vs 9.4%, p = 0.008 (Table 3).

### Predictors of severe disease

On multivariable logistic regression, age less than 1 month, moderate or severe underweight for age, hospitalization in the 28-day period preceding *E*. *coli* BSI, having an underlying chronic illness other than HIV infection and BSI without a definable focus were predictors of severe disease (Table 5). However, when the 12 recurrent *E*. *coli* BSI episodes were removed from the dataset and the logistic regression model repeated, only age less than 1 month, aOR 3.0 (95% CI: 1.6–5.7), hospitalization in the 28-day period preceding *E*. *coli* BSI, aOR 2.9 (95% CI: 1.6–5.3) and BSI without a definable focus, aOR 2.8 (95% CI: 1.4–5.9) remained statistically significant. In both logistic regression models, ESBL-ECBSI was not associated with severe disease.

**Table 5:** Predictors of severe infection determined by univariate and multivariable logistic regression analyses^1^.

Variable	Crude Odds ratio (95% CI)	Adjusted Odds ratio (95%CI)
Less than 1 month of age	2.1 (1.2–3.7)	2.6 (1.4–5.0)
Moderate or severe underweight for age^2^	2.2 (1.3–3.7)	2.0 (1.1–3.4)
Hospitalization during the 28-day period preceding *E*. *coli* BSI	3.0 (1.8–5.1)	2.7 (1.5–5.0)
Underlying chronic disease other than HIV infection	2.6 (1.4–5.0)	2.0 (1.0–4.2)
Hospital-acquired *E*. *coli* BSI	2.2 (1.3–3.7)	1
BSI with no definable focus	4.3 (2.1–8.7)	3.5 (1.7–7.1)
ESBL-producing *E*. *coli* BSI	2.3 (1.4–3.9)	1

^1^Predictors of severe infection was evaluated using data from all 455 *E*. *coli* BSI episodes

^2^Weight-for age z-score <-2 to >-3 standard deviations and <-3 standard deviations respectively; 95% CI = 95% confidence interval

## Discussion

The present study provides a more detailed description of the clinical and microbiological aspects of *E*. *coli* BSI at our institution than a previous BSI study.[4] The overall incidence risk of *E*. *coli* BSI of 2.7 episodes / 1000 hospital admissions was lower than the incidence risk of *Klebsiella pneumoniae* BSI of 3.08 episodes / 1000 hospital admissions estimated over a 5-year period from 2006 to 2011.[11] Classification of BSI according to the route of acquisition revealed further differences between *E*. *coli* and *K*. *pneumoniae* BSI at our institution. Whilst more than 80% of *K*. *pneumoniae* BSI episodes were hospital-acquired, the results of the present study showed that only 28% of *E*. *coli* BSI episodes were hospital-acquired. Furthermore, 52 and 18 in every 100 ESBL-ECBSI episodes in the present study were hospital-acquired or community-acquired respectively, compared to the previous *K*. *pneumoniae* study which showed that 92 and 2 in every 100 ESBL *K*. *pneumoniae* BSI episodes were hospital acquired or community-acquired, respectively.[11] Recent studies suggest that ESBL-producing *K*. *pneumoniae* is more easily transmitted than ESBL-producing *E*. *coli* in hospital settings, which could partially explain the differences observed between these two Enterobacteriaceae infections.[26,27]

Risk factors for ESBL-ECBSI included hospitalization in the preceding 28-day period, a temperature of ≥38° Celsius at the time of *E*. *coli* BSI, residency in an intermediate care facility, underlying chronic illness and exposure to piperacillin-tazobactam in the preceeding 12 months. When the multivariable regression model was re-run without the 12 recurrent *E*. *coli* BSI episodes, residency in an intermediate facility was no longer a statistically significant risk factor for ESBL-ECBSI. Although exposure to several antibiotics / antibiotic classes in the 12-month period preceding *E*. *coli* BSI were highly significant on univariable analyses, only exposure to piperacillin-tazobactam remained statistically significant in the multivariable regression models. Approximately two thirds and 30% of the ESBL-ECBSI and non-ESBL-producing *E*. *coli* BSI episodes respectively, were preceded by multiple antibiotic exposures. However, prior exposure to 2 or more antibiotics / antibiotic classes was not shown to be a causal risk factor. These results suggest that perhaps exposure to piperacillin-tazobactam may not be a true causal risk factor but rather a marker of multiple antibiotic exposures. Therefore, collinearity between multiple antibiotic classes may explain the exclusion of many of the antibiotics/antibiotic classes from the multivariable regression models in Table 1. The causal relationship between specific antibiotic exposures and ESBL-ECBSI was thus difficult to tease out in this study because detailed information about these exposures were not captured, including the total exposure time of each antibiotic in the 12-month period preceding *E*. *coli* BSI. Hospitalization in the preceding 28-day period and having an underlying chronic illness other than HIV infection remained significant risk factors in all multivariable regression models evaluated, suggesting that these variables are likely to be true causal risk factors for ESBL-ECBSI in our patient population. Both of these factors allow for increased contact between sick and/or convalescing children, and hence may create opportunities for the transmission of resistant Enterobacteriacae and plasmid-mediated resistance genes between patients.[9]

In the present study *E*. *coli* BSI mainly manifested without a definable clinical focus. When a site of infection was evident, the urinary tract was primarily involved and to a lesser extent the gastrointestinal tract. A French study showed that the urinary and gastrointestinal tracts were the main portals of entry of *E coli* strains that cause BSI in children and adolescents less than 18 years. Furthermore, differential expression of microbial virulence factors identified in that study may in part explain differences in the portal of entry of *E*. *coli* strains.[1]

According to the antibiotic susceptibility profile of non-ESBL *E*. *coli* isolates, 88% and 98% of the non-ESBL *E*. *coli* strains were susceptible to combinations of ampicillin and gentamicin, and piperacillin-tazobactam and amikacin respectively. These antibiotic combinations are currently recommended as empiric therapy for community-acquired and hospital-acquired BSI, respectively at RCWMCH. However, 57% and 8.8% of the non-ESBL *E*. *coli* BSI episodes in the present study were treated with either 3^rd^ generation cephalosporins or carbapenems respectively, suggesting that these antibiotic classes could have been used inappropriately to treat many of the non-ESBL *E*. *coli* BSI episodes. It is hoped that, moving forward, an antibiotic stewardship programme, commenced at RCWMCH in mid-2012, should assist in improving antibiotic practice and limiting inappropriate use of these broad-spectrum agents.

In our study, mortality was higher in patients with ESBL-ECBSI treated with piperacillin-tazobactam and amikacin compared to a carbapenem as definitive therapy. The findings of a randomized clinical trial that determined whether piperacillin-tazobactam was as effective as meropenem for treating BSI caused by *E*. *coli* or *K*. *pneumoniae* with non-susceptibility to third generation cephalosporins was recently published. The mortality at 30 days after randomization, the study’s primary outcome measure was significantly higher in the piperacillin-tazobactam group, possibly suggesting that piperacillin-tazobactam may no longer be the recommended definitive therapy in this context.[28]

A French study previously showed that age younger than 3 months and non-urinary BSI were significantly associated with severe disease.[1] Our analysis in part agreed with these findings in that on multivariable logistic regression analysis we identified age less than 1 month and BSI without a definable focus of infection as predictors of severe disease. Furthermore, we identified an additional factor that may be contributing to severe disease in our setting, namely recent hospitalization. In our study, ESBL-ECBSI was not shown to be associated with severe disease. This finding is in agreement with the results of the French study, which showed that resistance to 3^rd^ generation cephalosporins was not a predictor of severe disease on multivariable logistic regression analysis.[1]

### Study limitations

The strength of this study is that it is one of the first large sub-Saharan African studies to describe *E*. *coli* BSI in children, providing clinical, epidemiological, and antibiotic susceptibility and therapy information on more than 400 BSI episodes. Due to the retrospective study design, there are limitations in the completeness and availability of clinical and laboratory information on some of the BSI episodes. The true burden of community-acquired *E*. *coli* BSI may have been underestimated due to the practice of administering broad-spectrum antibiotics to sick children at primary and secondary facilities before they are referred to our hospital, and this may have negatively impacted the positive yield from blood cultures. This practice is consistent with the WHO Integrated Management of Childhood Illness guidelines that have been implemented in South Africa.[29] Subsequent to the study period, the Centers for Diseases Control and Prevention, Atlanta revised its nomenclature, replacing the terms hospital-acquired, community-acquired and healthcare-associated infection with infection present on admission and healthcare-associated infection.[30–31] We retained the outdated classification in the current study because it permitted us to compare the results of the present study with those of our previous study on *K*. *pneumoniae* BSI. Since the bacterial isolates associated with the *E*. *coli* BSI episodes in the present study were not stored we were unable to confirm the presence of genes that confer extended spectrum beta-lactamase resistance. Consequently, cephalosporin resistance of some of the isolates may have been caused by Ambler class C (AmpC) beta-lactamases.[32]

In our study risk factors for ESBL-ECBSI were explored by using a control group comprising children with non-ESBL *E*. *coli* BSI. This is probably not the ideal comparator group because the source population from which children with ESBL-ECBSI originated differed from the sources of children with non-ESBL *E*. *coli* BSI. In our study, 82% of ESBL-ECBSI episodes were hospital-acquired or healthcare-associated infections suggesting that the predominant source population comprised hospitalized children and those with recent healthcare contact. By contrast, 56% of the non-ESBL *E*. *coli* BSI episodes used as the control group were community-acquired infections with no recent hospitalization or healthcare contact (Table 3). When individuals with infections caused by antibiotic-susceptible organisms are used as control-patients, the relationship between case-patients and certain risk factors, particularly antibiotic exposures may be biased resulting in the incorrect identification of an antibiotic as a risk factor because of inflation of the measure or effect or odds ratio.[33, 34] In our study, although piperacillin-tazobactam was shown to be a risk factor for ESBL-ECBSI the significance of this variable is doubtful because of concern of collinearity between multiple antibiotic exposures.

The low number of deaths meant that we were unable to define risk factors associated specifically with death, but instead were able to explore risk factors for severe disease, a composite variable of ICU admission and/or death.

## Conclusion

The current study extends our understanding of *E*. *coli* BSI at our institution. While the risk of *E*. *coli* BSI was shown to be lower than that of *K*. *pneumoniae* BSI, it remains an important cause of BSI at our hospital. This study suggest ESBL-producing *E*.*coli* BSI is rapidly increasing despite decline in the overall incidence of *E*.*coli* BSI and may have important impacts on clinical outcomes. This has implications for antibiotic management and prevention of transmission of resistant organisms between patients. Further prospective research is required in our setting, to explore the spectrum of ESBL-producing genes associated with cephalosporin-resistance as well as genetic virulence determinants of *E*. *coli* BSI, and to identify risk factors for ESBL-ECBSI using methodological approaches that limit selection bias such as the case-case control study design and control-patients without *E*. *coli* infection but from the same base population as patients with ESBL-ECBSI.[34].

## Supporting information

S1 File Data set for graphs on incidence. (XLSX)

S2 File Data set for 455 cases analyzed. (XLSX)

S3 File Data set for 443 cases analyzed without 12 records. (XLSX)
